# Orthopedic Tourism and Volunteerism: Joint Effort or Disjointed Mobility?

**DOI:** 10.1016/j.artd.2021.06.016

**Published:** 2021-07-19

**Authors:** David O’Sullivan, John P. McCabe, Gerard T. Flaherty

**Affiliations:** a Department of Orthopaedic Surgery, Galway University Hospitals, Galway, Ireland; b School of Medicine, National University of Ireland Galway, Galway, Ireland; c School of Medicine, International Medical University, Kuala Lumpur, Malaysia

**Keywords:** Orthopaedic tourism, Medical tourism, Medical travel, Volunteering, Surgical education

## Introduction

The availability and accessibility of international orthopedic care have increased dramatically over recent decades [1]. A prominent facet in the growth of this specialty has been the travel of medical tourists abroad to avail of orthopedic procedures, including arthroplasty. Concurrently, orthopedic clinicians may choose to travel abroad to deliver care to patients in resource-poor settings. This article considers orthopedic surgical travel from the dual perspective of orthopedic surgery and travel medicine. It will discuss the risks, benefits, and opportunities associated with the globalization of orthopedic surgery.

## Travel overseas for orthopedic care

The act of traveling across international borders in pursuit of healing is as old as civilization itself, with travel to India during ancient times for Ayurvedic treatments giving way to the practice among the middle classes of nineteenth century Europe of visiting spa destinations to “take the waters,” often for the purpose of relieving chronic arthralgia [2]. The medical tourism industry has burgeoned in recent decades, with an estimated 30 to 50 million individuals traveling overseas each year for medical treatment, accounting for 35%-45% of all international inpatients receiving medical care [3]. Economic, sociocultural, and technological globalization have facilitated the growth of medical tourism. The motivations of bariatric, cosmetic, transplant, fertility, and stem cell tourists vary, but the greatly reduced costs of procedures performed in developing countries, prohibitive domestic waiting lists, and accessibility of treatment are common driving factors [4]. The availability of inexpensive flights and the internet have also been instrumental. The modern trend is for medical tourists to travel from more developed to developing countries, with diasporic medical tourists being particularly inclined to seek out medical care in countries with which they share a birth or ancestral link and from clinicians who have trained in developed countries [5]. Developed countries themselves remain popular as destinations for high-quality specialized medical care for wealthy patients traveling from abroad.

The elective nature of many orthopedic procedures, coupled with their high cost in developed economies, account for the growing importance of orthopedic tourism [4]. Medical tourists take a consumer-led approach to their health care, and they initiate their own care by seeking out providers and tourist packages that meet their needs. India and Thailand are widely recognized as international centers for affordable orthopedic surgery tourism. The cost of a hip arthroplasty in India is approximately one-fifth of that in the United States, for example (Table 1). Orthopedic tourists mostly travel to undergo hip and knee arthroplasty, but arthroscopy, joint resurfacing, spinal laminectomy, and spinal decompression are also frequently performed [4].

**Table 1:** Comparative cost of orthopedic procedures in surgical tourism destinations [3].

Country/Procedure	Hip arthroplasty (USD)	Knee arthroplasty (USD)
USA	47,000	48,000
India	9000	8500
Thailand	12,000	10,000
Singapore	11,000	13,000
Malaysia	10,000	8000
Mexico	17,300	14,650
Poland	6120	6375

USD, US dollars.

The lack of an accredited international registry limits our understanding of the true volume, movement patterns, and complications associated with orthopedic tourism. The international branch of the US-based Joint Commission, a not-for-profit organization, accredits medical services around the world, but many medical tourism services operate outside its remit [6]. Moreover, without the gatekeeper role of normal clinical referral pathways, orthopedic tourists are vulnerable to misleading or incomplete information from medical tourist agency or clinic websites. These typically present surgical procedures as routine and low-risk in nature and highlight the ancillary tourist attractions available to patients as vacation packages [7]. The lack of holistic pretravel evaluation of orthopedic tourists compromises their capacity to provide informed consent to their surgical procedure [8]. Confidentiality of personal medical data is a concern, and there is a risk that travellers are unaware of the potential for their information to be sold on to other medical service companies.

There is a paucity of authoritative evidence regarding the quality of care and clinical outcomes of procedures performed on orthopedic tourists. A systematic review of surgical tourism by Foley et al. reported complication rates as high as 56% including wound infection, poor functional outcome, and adverse cardiovascular events [4]. The risk of importation of antibiotic-resistant bacterial strains associated with inadequate antimicrobial stewardship represents a significant risk to surgical tourists and has public health implications in their home countries [8]. Long haul travel-related venous thromboembolism is a further perioperative complication of surgical tourism. It is unknown what proportion of medical tourists attend a travel clinic for specialized pretravel health information, including travel vaccines and malaria chemoprophylaxis, but advice to do so is rarely issued by medical tourism agencies via their websites [7]. Obese patients are overrepresented among orthopedic tourists and face unique travel health risks requiring careful multidisciplinary management [9].

Judicious patient selection and medical optimization are key determinants of the outcome of orthopedic surgical procedures. Both facets of care are at risk of being compromised in the context of orthopedic tourism. The lack of continuity of care and multidisciplinary preoperative care and follow-up by medical tourism providers, as well as the medicolegal complexities at play in foreign jurisdictions in cases where surgical negligence is alleged, should be carefully considered by prospective orthopedic tourists. While insurance policies do exist that cover surgical procedures performed in different jurisdictions and domestic health-care costs arising from complications in returning tourists, it is likely that many orthopedic tourists, in line with other international travellers, do not purchase such insurance [10]. Whether orthopedic tourists willingly disclose details of their overseas procedure to their primary care provider is also in doubt and has implications for their continuity of care.

The impact of orthopedic tourism on host countries also deserves our attention. While some national governments, such as the Malaysian government, actively promote their medical tourism industries abroad [11], the possibility exists in many popular medical tourist destinations that resources become diverted from the public health system to private hospitals serving wealthy tourists, thus generating a two-tiered health system [12]. The positive benefits of an international medical education may be undermined by the return to their countries of origin of well-trained specialists who choose to work exclusively in the lucrative private sector. Thus, medical tourism can inadvertently give rise to a brain drain of medical talent within countries with an already ailing public health infrastructure.

## Volunteerism in orthopedic surgery

Internationalization of orthopedic surgery involves not only the movement of patients but also of practitioners. Surgical volunteerism, or short-term surgical trips, is a term used to describe teams from developed countries embarking on “surgical missions” to low- and middle-income countries (LMICs) to deliver high-quality surgical care that is otherwise unavailable to local patients. The nature of the trip varies depending on the primary clinical needs in the region. Orthopedic expeditions range from acute trauma care necessitated by major disasters to missions where elective joint arthroplasty provides the main focus of the surgical travellers. The successful establishment of organizations such as Operation Rainbow and Orthopedics Overseas reflects the success of orthopedic surgeons’ efforts to provide care in LMICs. The major earthquake that struck Haiti in 2010 resulted in 150,000 deaths and 300,000 people injured, inspired over 500 American Academy of Orthopedic Surgeons and Orthopedic Trauma Association volunteer surgeons. Organizations such as Partners in Health were instrumental in coordinating the acute trauma care response [13]. Operation Walk, a global elective orthopedic volunteering initiative, has operated on over 17,000 patients in 20 countries at no cost to patients. The burden of illness attributed to orthopedic conditions in LMICs is considerable; consequently, a definite global need for orthopedic surgical volunteerism exists [14].

The successful execution of an orthopedic surgical mission is challenging and requires methodical preparation. First, the safety of the surgical team is of paramount importance. Volunteers are exposed to multiple risks including threats to personal safety and infectious diseases. Due diligence in preparation for the trip includes appropriate travel vaccination and malaria chemoprophylaxis where the risk demands it and familiarization with the region and meticulous planning with host hospital groups to implement security measures and provide adequate accommodation. The procurement of sustainable equipment must also be arranged [15]. A unique aspect of orthopedic volunteerism that requires consideration before embarking on a mission is the reliance on prosthetic implants. The challenging process of procuring and transporting equipment has been addressed by foundations such as Orthopedic Link through the establishment of supply chains involving the donation by implant manufacturers in high-income countries of surplus inventory to LMICs. Ethical aspects of implant donation that deserve consideration include minimum standard requirements of implants and potential financial incentives for manufacturers to donate surplus stock. Training and planning before the trip will ensure surgical teams are better prepared for the challenges they face when delivering care in resource-poor settings.

International volunteerism is fraught with ethical and legal considerations, and these must be addressed before any orthopedic mission. Historically, the “blitz surgery” model aimed to maximize the number of operative procedures performed. However, this approach led to a high rate of adverse clinical outcomes owing to a lack of continuity of care, inadequate supplies, and surgeons performing operations outside their scope of expertise. The development of guidelines for international surgical volunteers has shifted the focus of missions to the education of local surgeons, appropriate patient selection, and continuity of care [16]. The legal status of volunteers varies by country and the nature of the mission. In emergency settings, certain countries such as Australia and Pakistan have liability exemptions for volunteers acting “in good faith.” Countries such as the Philippines require all medical volunteers to purchase liability insurance, and volunteers and host organizations are responsible for all adverse outcomes incurred [17]. This evolution and regulation of orthopedic missions is a welcome development that recognizes the adaptations that must be made when delivering care in developing countries.

The globalization of orthopedic care has created educational opportunities for medical students, orthopedic trainees, and surgeons in both receiving and visiting countries. Establishing residential placements overseas in resource-poor settings may help orthopedic trainees deal with occupational burnout and expose them to a variety of unfamiliar orthopedic presentations. However, it is important that a symbiotic relationship is nurtured in which trainees are actively involved in educating health-care workers in their host hospital [18]. Medical student electives, organized in conjunction with orthopedic missions, would expose students to the challenges clinicians face in these settings and provide them with a more comprehensive view of global health.

## Conclusions

Governance of orthopedic practice pertaining to orthopedic tourism and orthopedic volunteerism is challenging. The COVID-19 pandemic has temporarily grounded many international travellers and dramatically reduced the number of elective orthopedic procedures being performed worldwide. The pandemic will undoubtedly have a negative impact on the movement of orthopedic tourists and on developing regions that depend on surgical volunteerism. The medical profession should advocate for international COVID-19 vaccine equity to prevent rising global health-care inequalities and ensure the continued success of globalized orthopedic care. While the potential for disjointed care exists for orthopedic tourists, the joint effort of volunteer orthopedic surgeons who travel to low-resource destinations to provide care is to be encouraged.

## Conflicts of interest

The authors declare that they have no known competing financial interests or personal relationships that could have appeared to influence the work reported in this article.

## Appendix A Supplementary data

Conflict of Interest Statement for O’Sullivan

Conflict of Interest Statement for Flaherty

Conflict of Interest Statement for McCabe
