# Supplementary material for: Orthopedic Tourism and Volunteerism: Joint Effort or Disjointed Mobility?
Source: Arthroplast Today. 2021 Jul 19;10:114–6. doi: 10.1016/j.artd.2021.06.016 (PMC8319507; doi:10.1016/j.artd.2021.06.016)
Supplement: Conflict of Interest Statement for Flaherty [file mmc2.docx]

# CONFLICT OF INTEREST STATEMENT

***American Association of Hip and Knee Surgeons***

(Adopted from the American Academy of Orthopaedic Surgeons disclosure statement)

The following form **must be filled out completely and submitted by each author (example, 6 authors, 6 forms).**

**All items require a response. If there is no relevant disclosure for a given item, enter "*None*.”**

Manuscript Title Title: **Orthopaedic tourism and volunteerism: joint effort or disjointed mobility?**

1. Royalties from a company or supplier (The following conflicts were disclosed) None

2. Speakers bureau/paid presentations for a company or supplier (The following conflicts were disclosed) None

3A. Paid employee for a company or supplier (The following conflicts were disclosed) None

3B. Paid consultant for a company or supplier (The following conflicts were disclosed) None

3C. Unpaid consultants for a company or supplier (The following conflicts were disclosed) None

4. Stock or stock options in a company or supplier (The following conflicts were disclosed) None

5. Research support from a company or supplier as a Principal Investigator (The following conflicts were disclosed) None

6. Other financial or material support from a company or supplier (The following conflicts were disclosed) None

7. Royalties, financial or material support from publishers (The following conflicts were disclosed) None

8. Medical/Orthopaedic publications editorial/governing board (The following conflicts were disclosed) **Prof. Gerard Flaherty serves as an unpaid Section Editor (non-communicable diseases) for the Journal of Travel Medicine.**

9. Board member/committee appointments for a society (The following conflicts were disclosed) **Prof. Gerard Flaherty serves as an unpaid Chair of the Publications Oversight Committee of the International Society of Travel Medicine.**

**Each author must sign AND print or type his/her name, date and submit a separate form**

In addition, one BLINDED Conflict of Interest form (no author names used) should be submitted per manuscript with all author disclosures.

**GERARD T. FLAHERTY**
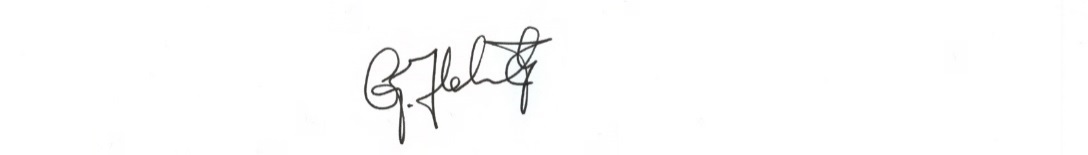
 **25 April 2021**

Author Name (Print or Type) Author Signature Date
